# Impact of Norepinephrine and Dopamine Infusion on Renal Arterial Resistive Index during Pre-Emptive Living Donor Kidney Transplantation: Propensity Score Matching Analysis

**DOI:** 10.3390/medicina60071066

**Published:** 2024-06-28

**Authors:** Jaewon Huh, Hyejin Kwon, Hunwoo Park, Sun Cheol Park, Sang Seob Yun, Min Suk Chae

**Affiliations:** 1 Department of Anesthesiology and Pain Medicine, Seoul St. Mary’s Hospital, College of Medicine, The Catholic University of Korea, Seoul 06591, Republic of Korea; 2 Department of Anesthesiology and Pain Medicine, Yeouido St. Mary’s Hospital, College of Medicine, The Catholic University of Korea, Seoul 06591, Republic of Korea; 3 Department of Surgery, Seoul St. Mary’s Hospital, College of Medicine, The Catholic University of Korea, Seoul 06591, Republic of Korea

**Keywords:** norepinephrine, dopamine, kidney transplantation

## Abstract

*Background:* Living donor kidney transplantation (LDKT) is a crucial treatment for end-stage renal disease, with pre-emptive LDKT (transplantation before dialysis initiation) offering significant benefits in graft function and patient survival. The selection of a vasopressor during LDKT, particularly between norepinephrine and dopamine, and its impact on renal arterial hemodynamics measured using the renal arterial resistive index (RARI) is poorly understood. *Methods:* This retrospective observational cohort study enrolled 347 eligible pre-emptive LDKT recipients from the Seoul St. Mary’s Hospital between January 2019 and June 2023. Utilizing propensity score matching (PSM), the patients were categorized into dopamine and norepinephrine groups to compare the effects of these vasopressors on the intraoperative RARI, postoperative estimated glomerular filtration rate (eGFR), and hourly urine output. The RARI was measured via the Doppler ultrasonography of the renal hilum and parenchyma post-graft vascular and ureteral anastomoses. *Results:* The preoperative differences in the recipients’ and donors’ characteristics were mitigated following PSM. The dopamine group exhibited higher intraoperative RARI values at the renal hilum (0.77 ± 0.11 vs. 0.66 ± 0.13, *p* < 0.001) and parenchyma (0.71 ± 0.1 vs. 0.6 ± 0.1, *p* < 0.001) compared to those of the norepinephrine group. However, these differences were not statistically significant on postoperative day 7. The norepinephrine infusion adjusted for the propensity scores was associated with significantly lower odds of an RARI > 0.8 (hilum: OR = 0.214, 95% CI = 0.12–0.382, *p* < 0.001; parenchyma: OR = 0.1, 95% CI = 0.029–0.348, *p* < 0.001). The early postoperative outcomes showed a higher eGFR (day 1: 30.0 ± 13.3 vs. 25.1 ± 17.4 mL/min/1.73 m^2^, *p* = 0.004) and hourly urine output (day 1: 41.8 ± 16.9 vs. 36.5 ± 14.4 mL/kg/h, *p* = 0.002) in the norepinephrine group. Furthermore, the long-term outcomes were comparable between the groups. *Conclusions:* Norepinephrine infusion during pre-emptive LDKT is associated with more favorable intraoperative renal arterial hemodynamics, as evidenced by a lower RARI and improved early postoperative renal function compared to those of dopamine. These findings suggest a potential preferential role for norepinephrine in optimizing perioperative management and early graft functions in LDKT recipients. Given the retrospective nature of this study, further prospective studies are needed to confirm these observations. Additionally, the study limitations include the potential for unmeasured confounding factors and the inability to determine causality due to its observational design.

## 1. Introduction

Living donor kidney transplantation (LDKT) is a well-established treatment for end-stage renal disease [1]. The recent guidelines recommend pre-emptive LDKT, wherein transplantation is performed before dialysis is initiated [2]. This approach has several benefits, including enhanced graft function and patient survival, as it avoids the complications associated with dialysis, such as cardiovascular diseases and infections [3,4,5,6]. In addition, pre-emptive LDKT offers improved patient welfare and lower societal costs [7,8,9,10]. However, patients who undergo it encounter challenges due to the rapid transition from end-stage renal disease to surgery, including volume imbalances and metabolic disturbances, such as hyperkalemia and abnormal calcium and phosphate levels [11,12].

The transplanted kidney undergoes altered autoregulation, relying more on blood flow than on blood pressure. High vascular resistance can result in hypertension, thereby reducing the blood flow and oxygen supply required for appropriate kidney function [13]. The use of vasopressors in renal transplantation is essential for maintaining an adequate blood pressure and ensuring the proper perfusion of the transplanted kidney. Norepinephrine and dopamine are commonly used vasopressors, each with distinct pharmacological properties. Although small doses of vasopressors can be used to manage low blood pressure, there is a lack of evidence regarding the optimal vasopressor for initial treatment [14,15,16].

Dopamine has been used for its potential renal-protective effects, which are believed to enhance renal blood flow and urine output. However, the recent studies suggest that dopamine may not provide significant renal benefits and could lead to an increased heart rate without substantial improvement in renal function. This raises concerns about its efficacy and safety in renal transplantation [17,18,19,20]. Norepinephrine is chosen for its strong α-adrenergic agonist properties and some β-adrenergic activity. It increases systemic vascular resistance and, to a lesser extent, cardiac output, which help maintain renal perfusion pressure. This is particularly important for transplanted kidneys that often cannot adequately respond to physiological and pharmacological stresses due to denervation. Norepinephrine’s ability to improve systemic hemodynamics and ensure adequate renal blood flow makes it a potentially superior choice for optimizing intraoperative and early postoperative renal function [21,22,23,24,25,26].

The renal arterial resistive index (RARI) is a critical tool used in Doppler ultrasonography to indirectly determine the resistance level within the renal and intrarenal vessels and assess the function of kidney grafts [27,28]. It is recognized as the most accurate ultrasound parameter for detecting kidney allograft dysfunction. The variability in RARI may be attributable to various factors, including intrarenal conditions (such as transplant rejection and acute tubular necrosis), extrarenal problems (such as ureteric obstruction and allograft compression), and systemic influences (such as the patient’s age and blood pressure fluctuations) [29]. Numerous studies have explored the relationship between the RARI and key outcomes such as allograft histology, acute rejection, and the potential risk of transplant failure, highlighting its importance as a diagnostic tool and a predictor of long-term transplant outcomes [30,31,32].

Limited studies have evaluated the effects of intraoperative norepinephrine and dopamine infusion on renal flow dynamics and early postoperative graft function recovery. In this study, we explored the effects of two vasopressor agents on the intraoperative RARI during pre-emptive LDKT. We also examined the influence of these drugs on the estimated glomerular filtration rate (eGFR) and hourly urine output post-transplantation.

## 2. Patients and Methods

### 2.1. Ethical Considerations

The protocol for this retrospective observational cohort study received approval from the Institutional Review Board and Ethics Committee of Seoul St. Mary’s Hospital (approval no. KC22RISI0395) on 7 June 2022. For the purposes of this study, authorization to access data was granted for the timeframe spanning from 7 June 2022 through 6 June 2023. Before initiating data collection, all the collected research data were anonymized (assigned random study numbers) to ensure the privacy of participants. This anonymization process was maintained throughout and after data collection for all analyses conducted, adhering to the principles outlined in the Declaration of Helsinki. Given the retrospective nature of the study, it was deemed unnecessary to obtain informed consent from the participants. We have reported our findings following the STROBE Statement guidelines to ensure clarity, transparency, and rigor in reporting observational studies.

### 2.2. Study Population

This study initially enrolled 700 adult patients (aged 19 years or older) who underwent elective pre-emptive LDKT at our hospital from January 2019 to June 2023. We excluded certain participants based on the following criteria, pediatric patients (younger than 19 years), those with a history of dialysis, individuals with atherosclerosis of the external iliac artery, cases involving grafts with multiple artery branches, patients receiving a graft on the right side due to differences in artery length between the left and right kidneys, recipients of deceased-donor or ABO-incompatible kidney transplants, patients undergoing multi-organ transplants that included the kidney, and patients requiring re-transplantation, which involves more complex surgical techniques. These complex techniques include multiple arterial anastomoses, vascular reconstruction, dual kidney transplantation, and transplantation in recipients with anatomical abnormalities. Additionally, we excluded patients with incomplete or missing data regarding the recipient and donor graft.

**Figure 1:**
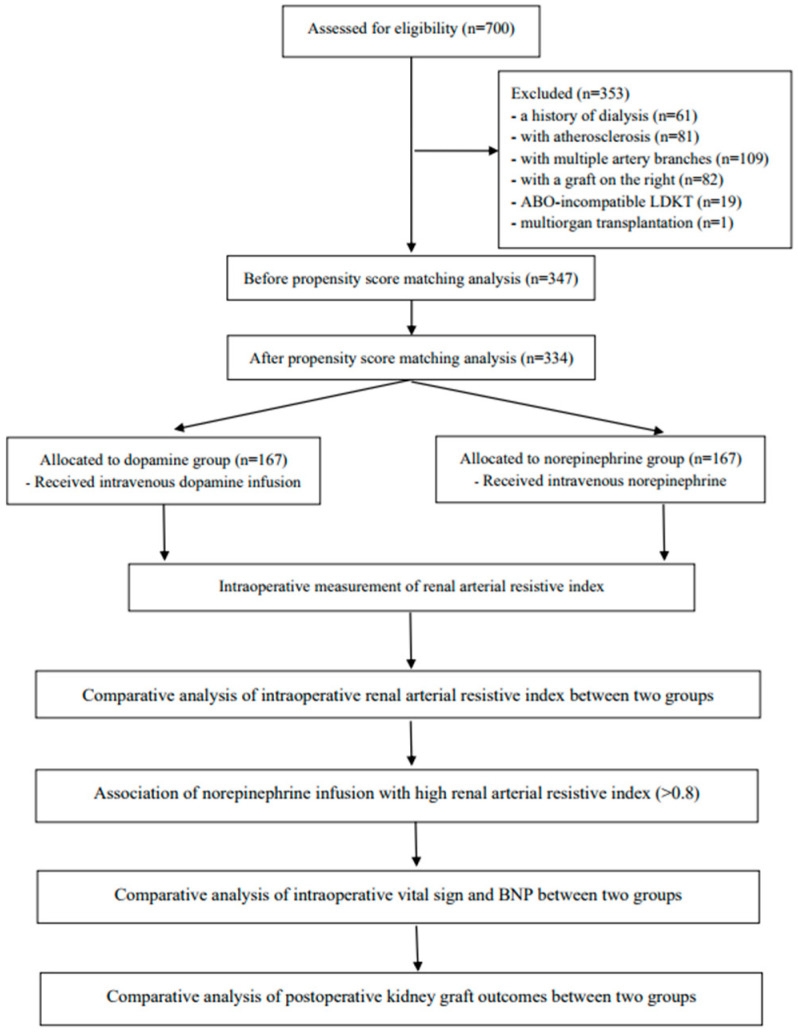
Study flow diagram.

Based on the aforementioned criteria, 353 patients were ineligible, and the remaining 347 patients were included in final analysis. Using propensity score matching (PSM), we identified 334 matched patients, categorizing them into dopamine (n = 167) and norepinephrine (n = 167) groups (Figure 1 and Supplemental File).

### 2.3. Surgery and General Anesthesia

For LDKT performed under general anesthesia [33], the surgical procedure commenced with a hockey stick-shaped (pararectal inverted J-shaped and curvilinear) incision to access the right pelvic fossa. Following the preparation of the graft on the back table, end-to-side anastomosis was performed, attaching the recipient’s external iliac artery and vein to the graft’s renal artery and vein using Prolene 6.0, a resorbable monofilament suture. Ureteroneocystostomy was performed using the Lich–Grègoir technique, which involved the insertion of a double-J stent. After ensuring meticulous hemostasis, reassessing the vascular anastomosis, and checking the renal pedicle area, closed drains were placed, and the wound was closed.

Balanced anesthesia was achieved using propofol and rocuronium for induction, whereas maintenance anesthesia was achieved using desflurane, medical air/oxygen, and the continuous infusion of remifentanil. The bispectral index was maintained at 40–50 to achieve the appropriate hypnotic depth. Mannitol was administered immediately before graft reperfusion. Crystalloids were administered to enhance urine flow and ensure optimal kidney graft perfusion, with the aim of achieving a target central venous pressure of 10–15 mmHg, or a hydration volume of 50–100 mL/kg.

### 2.4. Norepinephrine and Dopamine Infusion during Surgery

The hemodynamic status of the patients was meticulously managed to maintain a mean arterial pressure (MAP) ≥ 65 mmHg, as determined by the attending anesthesiologist’s discretion. This was achieved through the administration of inotropic infusions, particularly norepinephrine (Dalim Biotech, Seoul, Republic of Korea) or dopamine (Reyonpharm, Seoul, Republic of Korea), delivered via a central venous line in accordance with the surgery schedule. For the patients in the norepinephrine group, treatment commenced with a low dose of 0.05–0.1 mcg/kg/min, which was subsequently adjusted according to ongoing blood pressure monitoring to achieve optimal hemodynamic parameters. Similarly, the patients in the dopamine group were started on a low dose of 1–3 mcg/kg/min, which was increased to a medium dose of 5–10 mcg/kg/min if needed.

### 2.5. Intraoperative RARI Measurement

**Figure 2:**
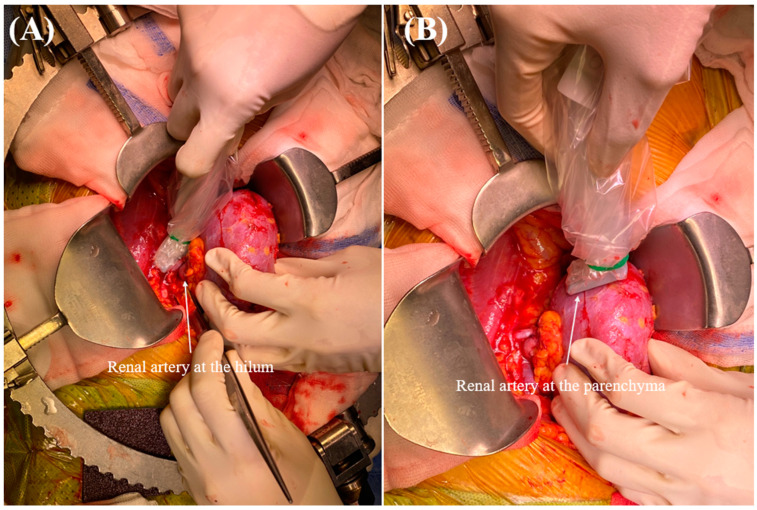
Doppler ultrasonography for renal arterial resistive index (RARI) measurement. RARI measurement at (**A**) the artery of the renal hilum and (**B**) the renal parenchyma.

**Figure 3:**
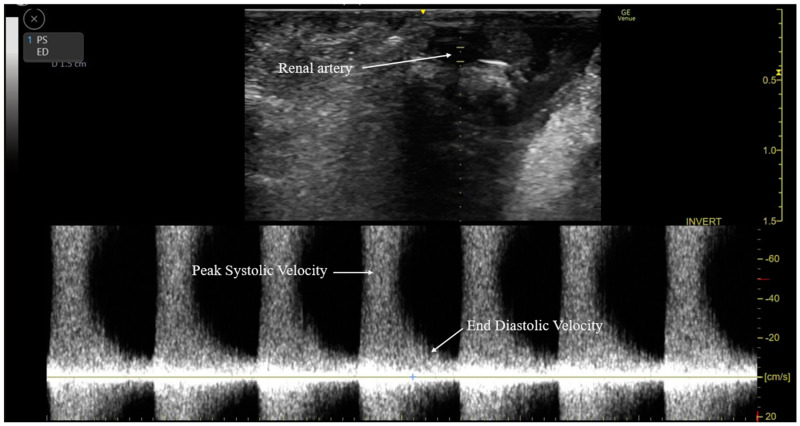
Measurement of renal arterial resistive index by using pulsed wave Doppler ultrasonography.

Following graft vascular and ureteral anastomosis, Doppler ultrasonography (Venue Go, GE Healthcare) was performed by the surgeon to measure the RARI and evaluate blood flow through the renal arteries at the renal hilum and parenchyma (Figure 2). The procedure began with the preparation of the ultrasound device, which was outfitted with a hockey stick-shaped transducer (L8-18i ultrasound transducer, GE Healthcare). Conductive gel was applied to the targeted areas on the renal artery and parenchyma to ensure the optimal transmission of ultrasound waves (pulsed wave Doppler) (Figure 3). The transducer was placed over the kidneys to locate the renal artery. The Doppler gate was positioned within the arterial lumen to capture optimal blood flow velocities during the cardiac cycle. The RARI was calculated as (peak systolic velocity − end-diastolic velocity)/peak systolic velocity [34]. This formula provides a dimensionless value indicative of the resistance in renal artery blood flow, with values > 0.8 defined as a high RARI [35]. The derived values were analyzed considering the clinical context of each patient, with all the pertinent findings and interpretations meticulously documented.

### 2.6. Clinical Variables for PSM

To ensure comparability between the dopamine and norepinephrine groups, we employed PSM based on a comprehensive set of preoperative, intraoperative, and donor graft factors. The preoperative recipient factors included sex, age, body mass index (BMI), and the presence of comorbidities such as diabetes mellitus (DM) and hypertension (HBP). Cardiac function markers such as ejection fraction and left ventricular mass index, along with systolic and diastolic blood pressures, heart rate, and various laboratory variables, including white blood cell count, neutrophil percentage, lymphocyte percentage, hemoglobin, albumin, electrolytes (sodium and potassium), creatinine, brain natriuretic peptide, high-sensitivity troponin I, corrected QT interval, and platelet count, were also considered. The intraoperative parameters included surgery duration, hourly fluid infusion rate, and the volume of blood loss. We also recorded serial intraoperative measurements of systolic, diastolic, and mean arterial pressures, heart rate, central venous pressure, and brain natriuretic peptide level. Donor and graft characteristics included donor sex (female), age, BMI, hemoglobin level, graft weight, and total ischemic time of the graft.

### 2.7. Postoperative Variables

The postoperative clinical factors included the RARI values recorded in the operating room and the ward, eGFR [36], hourly urine output, the need for rescue dialysis therapy, and the duration of intensive care unit (ICU) and hospital stays.

### 2.8. Statistical Analysis

The Shapiro–Wilk test was used to assess the normality of the distribution of continuous variables. The normally distributed variables are presented as means with standard deviations (SDs), whereas the non-normally distributed variables are presented as medians with interquartile ranges. The categorical variables are presented as numbers with percentages. To mitigate the effects of potential confounding factors on differences between groups, PSM was performed based on propensity scores, matching the patients on a one-to-one basis using greedy matching algorithms without replacement. The perioperative recipient and donor graft factors were compared between groups using the Mann–Whitney *U* test for continuous variables and the *χ*^2^ test or Fisher’s exact test for categorical variables as appropriate. The influence of intraoperative norepinephrine use on the intraoperative RARI was evaluated using multivariable logistic regression analysis after adjusting for propensity scores. The results are presented as odds ratios (ORs) with 95% confidence intervals (CIs). All statistical tests were two-sided. *p*-values < 0.05 were considered indicative of statistical significance. Statistical analyses were performed using R (version 2.10.1; R Foundation for Statistical Computing, Vienna, Austria) and SPSS for Windows (version 24.0; IBM Corp., Armonk, NY, USA).

## 3. Results

### 3.1. Demographic Characteristics

Of the 347 study participants, 173 (49.9%) were female. The mean age of the recipients was 49.7 ± 11.9 years, and the mean BMI was 23.5 ± 4.0 kg/m^2^. DM and HBP were present in 119 (34.3%) and 161 (46.4%) of the recipients, respectively. The mean serum level of creatinine prior to transplantation was 7.9 ± 2.7 mg/dL, and the mean hourly urine output was 2.1 ± 1.3 mL/kg/h. Among the living donors, 173 (49.9%) were female. The donors had a mean age of 48.4 ± 12.8 years and a mean BMI of 24.1 ± 3.1 kg/m^2^. The mean ischemic duration of the grafts was 58.8 ± 18.5 min, and the mean graft weight was 181.4 ± 39.7 g.

### 3.2. Comparison of Perioperative Factors before and after PSM

Before PSM, significant differences were observed between the two groups in terms of the preoperative characteristics of the recipients, particularly in the left ventricular mass index and hemoglobin level, as well as in the donors’ age (Table 1). However, after applying PSM to match the patients in the groups based on the aforementioned and potentially other relevant factors, the previously significant differences in perioperative recipient characteristics and donor graft parameters were rendered nonsignificant.

### 3.3. RARI in the Dopamine and Norepinephrine Groups in PS-Matched Patients

The RARI at the renal hilum and parenchyma were higher in the dopamine group than that in the norepinephrine group (Table 2). In addition, the proportion of patients with a high RARI (>0.8) was higher in the dopamine group than it was in the norepinephrine group. However, on postoperative day 7, these differences were not significant.

### 3.4. Association between Norepinephrine Infusion and a High RARI during Pre-Emptive LDKT

Norepinephrine infusion was significantly associated with a lower likelihood of a high RARI during pre-emptive LDKT, even after adjusting for the propensity scores. Specifically, the adjusted odds of achieving an RARI > 0.8 were significantly lower with norepinephrine infusion both at the renal hilum (adjusted OR = 0.214, 95% CI = 0.12–0.382, *p* < 0.001) and at the renal parenchyma (adjusted OR = 0.1, 95% CI = 0.029–0.348, *p* < 0.001) (Table 3).

### 3.5. Intraoperative Vital Signs and Brain Natriuretic Peptide Level in PSM Patients

During the surgical procedure, the heart rate observed after vascular graft and ureteral anastomosis, as well as at the conclusion of the surgery, was higher in the dopamine group than that in the norepinephrine group (Table 4). However, the other vital signs, including blood pressure and respiratory rate, along with the brain natriuretic peptide level, were comparable between the two groups.

### 3.6. Postoperative Kidney Graft Outcomes in PSM Patients

On the first postoperative day, both the eGFR and hourly urine output were higher in the norepinephrine group than that in the dopamine group (Table 5). However, subsequent evaluations of the postoperative variables, including eGFR, hourly urine output, the need for rescue dialysis therapy, and the duration of ICU and hospital stay, revealed no significant differences between the two groups.

## 4. Discussion

The novelty of our study lies in its focus on the intraoperative use of norepinephrine and dopamine during pre-emptive LDKT and their impact on renal arterial hemodynamics measured using the RARI. This topic has not been extensively explored in the literature. Our study utilizes PSM to minimize selection bias, ensuring a more accurate comparison between the effects of these two vasopressors. By demonstrating that norepinephrine is associated with more favorable intraoperative renal arterial hemodynamics and improved early postoperative renal function compared to those of dopamine, our findings provide critical insights that can enhance perioperative management in LDKT recipients.

We found that intraoperative norepinephrine infusion is more effective than dopamine in improving renal arterial hemodynamics during pre-emptive LDKT, as measured using the RARI at the renal hilum and parenchyma. After PSM, the norepinephrine infusion was associated with a significant decrease in the OR for a high RARI (>0.8) at the renal hilum (OR = 0.214) and parenchyma (OR = 0.1). While both norepinephrine and dopamine were capable of preventing intraoperative hypotension, the dopamine infusion was linked to an increased heart rate, an effect not observed with the norepinephrine infusion. In terms of postoperative kidney graft function, the patients receiving norepinephrine demonstrated a higher eGFR and hourly urine output on the first postoperative day. However, by the seventh postoperative day, outcomes such as the RARI, eGFR, hourly urine output, the need for rescue dialysis therapy, and the duration of ICU and hospital stays were similar between the two groups. Our findings suggest that a norepinephrine infusion may be a preferable option for maintaining systemic and renal hemodynamics during surgery and for early postoperative recovery, particularly when the denervated kidney allograft may impair the hemodynamic response to sympathomimetics.

Norepinephrine, which has strong α-adrenergic agonist properties and some β-adrenergic activity, plays a crucial role in increasing systemic vascular resistance, and to a lesser degree, cardiac output. Its ability to maintain renal perfusion pressure is particularly crucial, especially for transplanted kidneys, which often cannot adequately respond to physiological and pharmacological stresses due to their diminished autoregulatory capabilities. The denervation of kidney allografts removes the normal sympathetic nervous system inputs, necessitating the use of agents such as norepinephrine to ensure sufficient hemodynamic support [21,37]. Despite the effectiveness of vasopressors in managing such conditions, the optimal first-line vasopressor remains unclear, largely due to a scarcity of direct evidence. Nonetheless, there is a consensus that carefully titrating vasopressor dosages to avoid hypotension is preferable, given that the risks associated with low blood pressure exceed those of potential renal vasoconstriction [13,14]. Ensuring that the intraoperative MAP remains above 70 mmHg during kidney transplantation is crucial to prevent delayed graft function, which is often associated with lower MAP levels. The clinical recommendations suggest aiming for an MAP of 80–110 mmHg to protect kidney function, particularly during the critical reperfusion phase when an inadequate MAP can exacerbate kidney damage [14,38]. While direct comparisons between the responses of denervated kidney grafts to norepinephrine and those with intact autonomic regulation are challenging, several studies have indicated improvements in renal perfusion with norepinephrine administration, albeit within a broad spectrum of acceptable blood pressure levels [18,21,22,39]. In patients with septic shock, increasing the MAP from 65 to 75 mmHg using norepinephrine significantly increases the urinary output and RARI [22]. However, raising the MAP beyond this level does not result in additional benefits. In a study that explored the effects of varying MAP levels induced by norepinephrine on renal blood flow, eGFR, renal oxygen consumption, and oxygenation, the patients with norepinephrine-dependent vasodilatory shock and acute kidney injury following cardiac surgery demonstrated improved renal oxygen delivery, eGFR, and oxygenation when the MAP was increased from 60 to 75 mmHg. However, further elevation to 90 mmHg using norepinephrine only increased the renal vascular resistance without further improving perfusion, filtration, or oxygenation [21].

In patients with systemic inflammatory response syndrome due to an allograft, sepsis, or other triggers, hypotension may persist despite aggressive fluid resuscitation, often necessitating the use of a vasopressor. Traditionally, dopamine is preferred for its perceived ability to preserve blood flow to key organs such as the kidneys, brain, heart, and digestive system. In addition, concerns exist regarding norepinephrine use due to its intense vasoconstrictive effects. However, the renal-protective properties of dopamine have been disproven, revealing that it can reduce the blood flow to the mucosal lining of the digestive tract [19,20,40,41]. Contrary to the initial concerns, norepinephrine demonstrates more significant positive effects on renal blood flow and urine production than dopamine. While dopamine increases the mean renal blood flow by 20% without affecting the overall hemodynamics, norepinephrine significantly increases the MAP, cardiac output, and coronary and renal blood flow. In one study, dopamine increased the urine output, but did not improve creatinine clearance, whereas norepinephrine significantly enhanced urine output compared to low doses of dopamine, without significantly altering creatinine clearance [18]. Moreover, the harmful effects previously attributed to norepinephrine have not been substantiated. In fact, some studies have demonstrated that at clinically relevant doses, norepinephrine can enhance the blood flow to organs and tissues in various diseases. The concerns regarding severe vasoconstriction with norepinephrine administration have largely been linked to direct renal artery infusion in animal models at doses far exceeding those used in clinical practice [42,43,44].

Albanèse et al. demonstrated that norepinephrine, at doses typically used in hospitals, can improve or maintain kidney function in patients with pathological vasodilation and those who are well hydrated, but have normal systemic vascular resistance [23]. The benefits of norepinephrine include increased cyclooxygenase-2 expression in the kidney, leading to the increased production of cyclooxygenase-2-derived prostaglandins, such as PGE_2_ and PGI_2_ [45,46,47,48]. These substances mitigate norepinephrine-induced renal vasoconstriction and promote the dilation of the afferent arterioles of the kidneys, supporting glomerular filtration [46,48,49]. In addition, an increase in systemic blood pressure can trigger renal vasodilation by reducing renal sympathetic tone via a baroreceptor feedback mechanism [42]. Albanèse et al. found that norepinephrine administered at an average dose of up to 1.3 ± 0.3 µg/kg/min effectively restored the MAP and urine production in patients with septic shock and oliguria [23]. This observation is in line with other clinical studies [50,51,52] and experimental models demonstrating that norepinephrine can restore kidney function and blood flow during endotoxemia [25,26,53]. While norepinephrine is effective for the treatment of septic shock, its safety in other clinical conditions is uncertain [54]. However, Albanèse et al. demonstrated that norepinephrine, even at doses up to 0.5 µg/kg/min, is safe and effective for increasing the MAP and cerebral perfusion pressure without causing kidney dysfunction in well-hydrated patients with head injuries [23]. This finding is in line with the results of Morimatsu et al., who demonstrated that norepinephrine can maintain kidney function in hypotensive post-cardiac surgery patients [24]. Our results further support the greater effectiveness of norepinephrine than that of dopamine in maintaining renal regional arterial inflow and parenchymal function during LDKT.

While norepinephrine has shown benefits in maintaining renal perfusion and supporting early postoperative graft function, it is important to consider its potential adverse effects. Norepinephrine, primarily through its α-adrenergic activity, can cause vasoconstriction, which might reduce the blood flow to other vital organs and tissues, potentially leading to ischemic complications. This is particularly concerning in patients with compromised vascular integrity. Additionally, norepinephrine can increase the cardiac afterload, which may exacerbate heart conditions in susceptible individuals. There is also a risk of tissue ischemia and gangrene, especially in the extremities, due to prolonged or high-dose infusion [21,55,56]. Therefore, the careful monitoring and titration of norepinephrine are crucial to balancing its benefits and risks during and after surgery.

The intraoperative ultrasound imaging of renal artery flow is notably effective for the early detection of acute kidney injury (AKI), boasting high sensitivity and specificity [57]. Suggested for use in hemodynamically stable patients before sternotomy by Kajar et al., transesophageal echocardiography has proven instrumental in linking increased RARI values to AKI post-coronary artery bypass graft surgery, with a 26% diagnosis rate among patients. Particularly, an RARI above 0.7 was associated with higher AKI rates, establishing an elevated RARI as an independent AKI predictor post-surgery [58]. This predictive capacity extends to non-cardiac procedures and sepsis, with an RARI greater than 0.7 indicating an AKI with high accuracy in orthopedic surgery patients [59]. In kidney transplantation, transit time flowmetry and graft arterial flowmetry have been employed to assess renal artery flow and the RARI, which are vital for forecasting the immediate and delayed graft functions. Some studies, including those by Król et al. and Pravisani et al., have demonstrated significant correlations between the RARI levels and graft function outcomes, underscoring the importance of the RARI in evaluating transplant success [60,61]. Our research, while not primarily focused on the RARI’s direct link to AKI, explored the effects of norepinephrine and dopamine on the RARI during surgery. Our findings reveal that a high RARI (>0.8) could adversely affect early graft function recovery, emphasizing the significance of RARI monitoring and management in the intraoperative phase to enhance postoperative results.

Our study had several limitations that must be acknowledged. First, we did not measure the graft renal artery length or inner radius in the operating room, which could have influenced the graft flow and renal flow outcomes in both the groups. To mitigate this, we exclusively selected left-sided kidneys for grafting to reduce the selection bias associated with arterial length differences between left and right kidneys. However, this choice limits the generalizability of our findings to right-sided grafts or grafts with multiple arterial branches. Second, we only included grafts with a single artery, preventing us from assessing the potential differences in drug responses in grafts with multiple arterial branches. Third, although atherosclerosis is prevalent among patients undergoing kidney transplantation, we were unable to determine the effects of the two drugs on the RARI in individuals with such vascular characteristics. Fourth, the retrospective study design inherently limits our ability to establish causality and control for all the confounding variables. While propensity score matching (PSM) was employed to minimize the selection bias, residual confounding cannot be entirely eliminated. Additionally, the inability to randomize patients may introduce a selection bias, as treatment decisions were based on clinical judgment rather than random assignment. Furthermore, due to the retrospective nature of the study, we could not standardize the doses of norepinephrine and dopamine or establish target blood pressure levels, which may have influenced the outcomes. Our study also excluded patients who had undergone dialysis, limiting the generalizability of our findings to this population. Lastly, we focused exclusively on pre-emptive LDKT, thereby excluding any potential hemodynamic changes in patients who had previously undergone dialysis. Despite these limitations, our study provides valuable insights into the comparative effects of norepinephrine and dopamine on renal arterial hemodynamics and early postoperative renal function during pre-emptive LDKT. Nonetheless, further prospective randomized controlled trials are necessary to verify our findings and enhance our understanding of the optimal vasopressor for kidney transplant patients.

## 5. Conclusions

Our investigation into the intraoperative use of vasopressors during pre-emptive LDKT reveals the superiority of norepinephrine over dopamine in enhancing renal arterial hemodynamics, as evidenced by a significantly lower OR for a high RARI. Our results suggest that norepinephrine can effectively maintain both systemic and renal hemodynamics, without increasing the heart rate associated with dopamine. Despite similar outcomes by the seventh postoperative day, norepinephrine use is associated with a higher eGFR and urine output in the immediate postoperative period, suggesting that is preferable for early graft function recovery. These findings, while highlighting the need for further prospective trials, provide valuable guidance for vasopressor selection in LDKT, enhancing postoperative management and patient care.

## Figures and Tables

**Table 1:** Comparison of perioperative factors between the dopamine and norepinephrine groups before and after propensity score matching.

	Before Propensity Score Matching				After Propensity Score Matching			
Group	Dopamine	Norepinephrine	*p*	SD	Dopamine	Norepinephrine	*p*	SD
n	177	170			167	167		
**Preoperative recipient factors**								
Sex (female)	85 (48.0%)	88 (51,8%)	0.486	0.075	81 (48.5%)	86 (51.5%)	0.584	0.06
Age (years)	49.9 ± 12.7	49.5 ± 10.9	0.734	−0.04	49.7 ± 12.9	49.5 ± 10.9	0.905	−0.014
Body mass index (kg/m^2^)	23.3 ± 3.9	23.7 ± 4.1	0.298	0.109	23.4 ± 3.9	23.8 ± 4.1	0.368	0.096
*Comorbidity*								
Diabetes mellitus	62 (35.0%)	57 (33.5%)	0.769	−0.032	59 (35.3%)	55 (32.9%)	0.644	−0.051
Hypertension	87 (49.2%)	74 (43.5%)	0.294	−0.113	83 (49.7%)	72 (43.1%)	0.227	−0.132
*Echocardiography*								
Ejection fraction (%)	61.1 ± 5.5	59.8 ± 7.0	0.043	−0.196	61.0 ± 5.5	60.0 ± 6.7	0.16	−0.136
Left ventricular mass index (g/m^2^)	129.9 ± 83.8	127.4 ± 44.2	0.728	−0.056	123.4 ± 36.2	127.6 ± 44.6	0.35	0.094
*Vital sign*								
Systolic blood pressure (mmHg)	133.7 ± 13.2	131.0 ± 13.5	0.059	−0.201	133.4 ± 13.2	130.9 ± 13.5	0.092	−0.183
Diastolic blood pressure (mmHg)	82.0 ± 9.8	81.0 ± 9.3	0.327	−0.108	81.8 ± 9.9	81.0 ± 9.3	0.437	−0.088
Heart rate (beats/min)	78.5 ± 9.2	78.6 ± 9.4	0.859	0.019	78.8 ± 9.3	78.6 ± 9.3	0.874	−0.017
Hourly urine output (mL/kg/h)	2.1 ± 1.4	2.0 ± 1.3	0.638	−0.052	2.1 ± 1.3	2.0 ± 1.3	0.799	−0.029
Brain natriuretic peptide (pg/mL)	245.0 ± 483.8	278.6 ± 718.4	0.61	0.047	226.6 ± 442.2	278.6 ± 723.6	0.429	0.072
High-sensitivity troponin I (pg/mL)	45.5 ± 71.0	62.3 ± 199.0	0.291	0.085	45.6 ± 72.6	46.4 ± 84.7	0.923	0.004
Corrected QT interval (ms)	451.1 ± 29.3	450.5 ± 31.0	0.843	−0.021	451.0 ± 29.5	450.2 ± 31.0	0.793	−0.028
*Laboratory variables*								
White blood cell count (×10^9^/L)	6.7 ± 3.1	6.8 ± 2.6	0.88	0.018	6.7 ± 3.2	6.8 ± 2.6	0.834	0.026
Neutrophil (%)	72.7 ± 13.1	71.4 ± 15.1	0.411	−0.083	72.2 ± 13.1	71.5 ± 15.2	0.633	−0.049
Lymphocyte (%)	17.8 ± 9.0	18.4 ± 9.0	0.59	0.058	18.0 ± 9.1	18.3 ± 9.0	0.748	0.036
Hemoglobin (g/dL)	10.7 ± 1.4	10.4 ± 1.4	0.034	−0.23	10.6 ± 1.4	10.4 ± 1.4	0.113	−0.173
Albumin (g/dL)	4.1 ± 0.4	4.0 ± 0.5	0.115	−0.163	4.1 ± 0.4	4.0 ± 0.5	0.239	−0.123
Sodium (mEq/L)	136.5 ± 3.9	137.2 ± 4.6	0.112	0.158	136.7 ± 3.7	137.2 ± 4.7	0.309	0.101
Potassium (mEq/L)	4.6 ± 0.5	4.5 ± 0.5	0.234	−0.125	4.6 ± 0.5	4.6 ± 0.5	0.322	−0.107
Chloride (mEq/L)	99.9 ± 5.6	100.4 ± 6.3	0.381	0.089	100.2 ± 5.6	100.4 ± 6.4	0.728	0.036
Platelet count (×10^9^/L)	182.3 ± 61.4	186.5 ± 66.2	0.544	0.063	183.3 ± 61.7	187.6 ± 66.1	0.546	0.064
International normalized ratio	1.1 ± 0.9	1.1 ± 0.3	0.583	−0.127	1.1 ± 0.9	1.1 ± 0.3	0.597	−0.129
Creatinine (mg/dL)	7.7 ± 2.7	8.1 ± 2.7	0.22	0.132	7.7 ± 2.7	8.1 ± 2.6	0.261	0.123
**Intraoperative recipient factors**								
Operation time (min)	226.3 ± 44.3	230.0 ± 43.2	0.432	0.085	227.6 ± 44.8	230.1 ± 43.5	0.601	0.058
Hourly fluid infusion (mL/kg/h)	9.6 ± 3.7	10.0 ± 3.2	0.247	0.133	9.7 ± 3.7	10.0 ± 3.2	0.469	0.086
Hemorrhage (mL)	171.1 ± 69.6	160.5 ± 66.8	0.151	−0.158	171.3 ± 68.6	160.4 ± 66.2	0.143	−0.162
**Donor and graft factors**								
Sex (female)	85 (48.0%)	88 (51.8%)	0.486	0.075	81 (48.5%)	86 (51.5%)	0.584	0.06
Age (years)	49.7 ± 13.0	47.0 ± 12.4	0.049	−0.217	49.1 ± 13.1	46.9 ± 12.5	0.117	−0.177
Body mass index (kg/m^2^)	24.1 ± 3.2	24.1 ± 3.1	0.894	0.015	24.1 ± 3.2	24.1 ± 3.1	0.944	0.008
Hemoglobin (g/dL)	13.8 ± 1.1	13.8 ± 1.2	0.917	0.011	13.8 ± 1.1	13.8 ± 1.2	0.903	0.013
Graft weight (g)	185.2 ± 41.3	177.4 ± 37.6	0.063	−0.21	183.9 ± 41.2	177.1 ± 37.8	0.12	−0.18
Total ischemic time (min)	57.7 ± 15.0	59.9 ± 21.5	0.261	0.104	58.3 ± 15.2	59.9 ± 21.7	0.434	0.074

**Abbreviations:** SD, standard deviation. Values are expressed as mean (standard deviation) and number (percentage).

**Table 2:** Comparison of the renal arterial resistive index between dopamine and norepinephrine groups in PS-matched patients.

Group	Dopamine	Norepinephrine	*p*
	167	167	
**In the operating room (after renal vascular and ureter anastomosis)**			
*Renal arterial resistive index*			
at renal hilum	0.77 ± 0.11	0.66 ± 0.13	<0.001
at renal parenchyma	0.71 ± 0.1	0.6 ± 0.1	<0.001
*Renal arterial resistive index (>0.8)*			
at renal hilum	66 (39.5%)	20 (12.0%)	<0.001
at renal parenchyma	25 (15.0%)	3 (1.8%)	<0.001
**In the ward (on postoperative 7)**			
*Renal arterial resistive index*			
at renal hilum	0.64 ± 0.11	0.64 ± 0.05	0.945
at renal parenchyma	0.56 ± 0.11	0.55 ± 0.05	0.382
*Renal arterial resistive index (>0.8)*			
at renal hilum	4 (2.4%)	0 (0.0%)	0.123
at renal parenchyma	3 (1.8%)	0 (0.0%)	0.248

**Abbreviations:** PS, propensity score. Values are expressed as median (interquartile) and number (proportion).

**Table 3:** Association between norepinephrine infusion and renal arterial resistive index > 0.8, adjusted for propensity scores during pre-emptive living donor kidney transplantation.

	*β*	Odds Ratio	95% CI	*p*
**In the operating room (after renal vascular and ureter anastomosis)**				
*Norepinephrine adjusted for PS*				
*Renal arterial resistive index (>0.8)*				
at renal hilum	−1.543	0.214	0.12–0.382	<0.001
at renal parenchyma	−2.301	0.1	0.029–0.348	<0.001

**Abbreviations:** CI, confidence interval; PS, propensity score.

**Table 4:** Comparison of intraoperative vital signs and the brain natriuretic peptide level among PS-matched patients from the dopamine and norepinephrine groups.

Group	Dopamine	Norepinephrine	*p*
	167	167	
**At the beginning of the surgery**			
Systolic blood pressure (mmHg)	126.5 ± 13.4	124.8 ± 9.8	0.185
Diastolic blood pressure (mmHg)	77.6 ± 11.0	75.4 ± 13.3	0.091
Mean blood pressure (mmHg)	93.9 ± 10.3	91.8 ± 11.0	0.075
Heart rate (beats/min)	73.2 ± 9.3	74.5 ± 7.4	0.158
Central venous pressure (mmHg)	5.6 ± 2.0	5.7 ± 1.8	0.841
Brain natriuretic peptide (pg/mL)	147.8 (94.8–251.2)	139.6 (81.9–295.9)	0.583
**After vascular graft and ureteral anastomosis**			
Systolic blood pressure (mmHg)	145.7 ± 6.9	144.5 ± 6.7	0.137
Diastolic blood pressure (mmHg)	80.0 ± 7.8	80.1 ± 6.9	0.888
Mean blood pressure (mmHg)	94.5 ± 10.1	93.5 ± 9.0	0.526
Heart rate (beats/min)	96.5 ± 10.5	79.9 ± 11.2	<0.001
Central venous pressure (mmHg)	11.7 ± 2.1	11.9 ± 3.0	0.425
**At the end of the surgery**			
Systolic blood pressure (mmHg)	132.9 ± 16.7	131.5 ± 15.6	0.428
Diastolic blood pressure (mmHg)	75.2 ± 10.6	74.9 ± 9.6	0.787
Mean blood pressure (mmHg)	94.5 ± 10.1	93.8 ± 9.0	0.526
Heart rate (beats/min)	96.2 ± 15.0	88.8 ± 14.2	<0.001
Central venous pressure (mmHg)	7.3 ± 2.9	7.4 ± 3.3	0.793
Brain natriuretic peptide (pg/mL)	119.0 (67.7–198.6)	101.5 (62.2–174.4)	0.14

**Abbreviations:** PS, propensity score. Values are expressed as mean (standard deviation) and median (interquartile).

**Table 5:** Comparison of postoperative kidney graft outcomes among PS-matched patients in the dopamine and norepinephrine groups.

Group	Dopamine	Norepinephrine	*p*
	167	167	
**Estimated glomerular filtration rate (mL/min/1.73 m^2^)**			
Postoperative day 1	25.1 ± 17.4	30.0 ± 13.3	0.004
Postoperative day 2	60.2 ± 30.0	60.8 ± 31.8	0.841
Postoperative day 3	70.7 ± 32.2	69.4 ± 32.1	0.702
Postoperative day 7	84.3 ± 34.3	80.9 ± 31.2	0.34
Hourly urine output (mL/kg/h)			
Postoperative day 1	36.5 ± 14.4	41.8 ± 16.9	0.002
Postoperative day 2	29.0 ± 11.9	29.3 ± 10.6	0.804
Postoperative day 3	26.4 ± 10.8	26.5 ± 9.5	0.949
Postoperative day 7	15.4 ± 5.8	15.3 ± 7.5	0.921
Rescue dialysis therapy	8 (4.8%)	11 (6.6%)	0.479
ICU stay (day)	2 (2–2)	2 (2–2)	0.206
Hospital stay (day)	13 (12–14)	13 (12–14)	0.476

Values are expressed as mean (standard deviation) and median (interquartile).

## Data Availability

Data are contained within this article and the Supplemental File.

## Supplementary Materials

The following supporting information can be downloaded at: https://www.mdpi.com/article/10.3390/medicina60071066/s1, Table S1: Inclusion and exclusion criteria.

## Abbreviations

RARI: renal arterial resistive index; LDKT: living donor kidney transplantation; ESRD: end-stage renal disease; PSM: propensity score matching; DM: diabetes mellitus; HBP: hypertension; eGFR: estimated glomerular filtration; BMI: body mass index; ICU: intensive care unit; AKI acute kidney injury.
