# Supplementary material for: Impact of Norepinephrine and Dopamine Infusion on Renal Arterial Resistive Index during Pre-Emptive Living Donor Kidney Transplantation: Propensity Score Matching Analysis
Source: Medicina (Kaunas). 2024 Jun 28;60(7):1066. doi: 10.3390/medicina60071066 (PMC11278998; doi:10.3390/medicina60071066)
Supplement: Supplementary file 1 [file medicina-60-01066-s001.zip › medicina-3056720-supplementary.pdf]

Supplemental Table S1. Inclusion and exclusion criteria

Inclusion criteria	Exclusion criteria
1. Adult patients aged ≥ 19 years<br>2. Elective preemptive living donor kidney transplantation<br>3. Availability of complete recipient and donor graft data	1. Patients aged < 19 years<br>2. History of dialysis<br>3. Atherosclerosis of the external iliac artery<br>4. Grafts with multiple arterial branches<br>5. Right-sided grafts due to differences in arterial length between the left and right kidneys<br>6. Deceased-donor or ABO-incompatible kidney transplants<br>7. Multi-organ transplants, including the kidney<br>8. Need for re-transplantation<br>9. Patients requiring more complex surgical techniques (multiple arterial anastomoses, vascular reconstruction, dural kidney transplantation, and transplantation in recipients with anatomical abnormalities)<br>10. Incomplete or missing data related to the recipient or donor graft
